# Emerging Trends in Snake Venom-Loaded Nanobiosystems for Advanced Medical Applications: A Comprehensive Overview

**DOI:** 10.3390/pharmaceutics17020204

**Published:** 2025-02-06

**Authors:** Álisson E. F. Alves, Anne B. C. Barros, Lindomara C. F. Silva, Lucas M. M. Carvalho, Graziela M. A. Pereira, Ana F. C. Uchôa, José M. Barbosa-Filho, Marcelo S. Silva, Karla P. O. Luna, Karla S. R. Soares, Francisco H. Xavier-Júnior

**Affiliations:** 1 Laboratory of Pharmaceutical Biotechnology (BioTecFarm), Department of Pharmacy, Federal University of Paraíba (UFPB), Campus I-Castelo Branco III., Joao Pessoa 58051-900, PB, Brazil; aefa@ltf.ufpb.br (Á.E.F.A.); annebcbarross@gmail.com (A.B.C.B.); lindomaracristina16@gmail.com (L.C.F.S.); lucas.medeiros2@academico.ufpb.br (L.M.M.C.); graziela.maria.dearaujo@gmail.com (G.M.A.P.); anaflaviauchoauf@gmail.com (A.F.C.U.); karllasamara@yahoo.com.br (K.S.R.S.); 2 Post-Graduated Program in Natural and Synthetic Bioactive Products, Federal University of Paraíba (UFPB), Campus I-Castelo Branco III., Joao Pessoa 58051-900, PB, Brazil; jbarbosa@ltf.ufpb.br (J.M.B.-F.); marcelosobral.ufpb@gmail.com (M.S.S.); 3 Venomics Laboratory (LabVenom), Center for Biological and Health Sciences, State University of Paraíba (UEPB), Campus I, Bodocongó, Campina Grande 58429-600, PB, Brazil; karlaceatox@yahoo.com.br

**Keywords:** drug delivery, drug discovery, nanomedicine, nanoformulation, venom peptides

## Abstract

Advances in medical nanobiotechnology have notably enhanced the application of snake venom toxins, facilitating the development of new therapies with animal-derived toxins. The vast diversity of snake species and their venom complexities underline the need for ongoing research. This review is dedicated to exploring the integration of snake venom with nanoparticles to enable their use in human therapies aiming to develop treatments. The complex mixture of snake venom not only inflicts significant pathological effects but also offers valuable insights for the creation of innovative therapies, particularly in the realm of nanobiotechnology. Nanoscale encapsulation not only mitigates the inherent toxicity of snake venom but also amplifies their antitumoral, antimicrobial, and immunomodulatory properties. The synergy between venom-derived macromolecules and nanotechnology offers a novel pathway for augmenting the efficacy and safety of conventional antivenom therapies, extending their applicability beyond treating bites to potentially addressing a myriad of health issues. In conclusion, nanotechnology presents a compelling therapeutic frontier that promises to improve current treatment modalities and ameliorate the adverse effects associated with venomous snakebites.

## 1. Introduction

Snakebite accidents represent a major public health problem worldwide [1,2]. However, it was only recently recognized as a neglected tropical disease by the World Health Organization (WHO) in 2017 [3]. Due to their physiology, snakes are abundant in warm climates, particularly in tropical regions with higher rates of snakebite accidents [4]. Many victims do not opt for healthcare services and instead resort to traditional treatments [5] and different needs are required in different communities [6]. Nevertheless, available data show that, annually, 4.5 to 5.4 million people are affected by snakebites. Of these, 1.8 to 2.7 million develop clinical illnesses, and 81,000 to 138,000 die due to the disease’s implications [3]. The WHO has subsequently developed a comprehensive plan known as the snakebite roadmap, aiming to achieve the ambitious objective of reducing the global burden of snakebites by 50% by the year 2030 [7].

Brazil is recognized for its vast biodiversity, of which Viperidae and Elapidae are snake families considered venomous and of medical importance [8,9,10]. Venomous and poisonous animals provide a wide variety of compounds, among which proteins, peptides, and neurotransmitters are the ones that draw the most attention for scientific research [11]. Nanotechnology has been applied for various purposes within the healthcare field with the production of nanomaterials. In the context of snakebite, nanoparticles have shown promising results in the development of more efficient therapies [12]. The encapsulation of snake venom in polymeric nanoparticles, as an example, is well studied due to its biodisponibility and selectivity for treatment, as well as the decrease in systemic toxicity [13].

**Figure 1:**
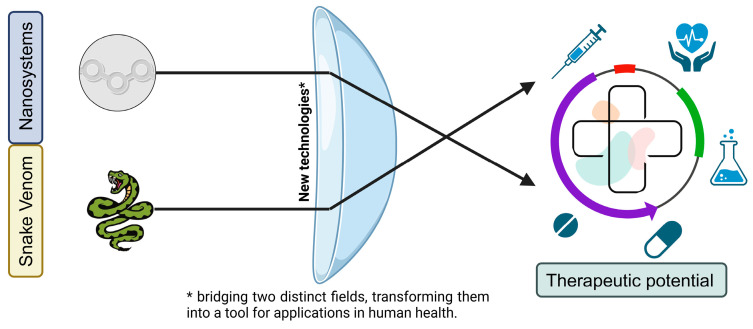
Uniting nature and technology for health purposes.

Within this context, the present review holds significant promise in elucidating the therapeutic potential of nanosystems in conjunction with snake venom, as documented in the existing literature (Figure 1).

## 2. Snake Venom Composition

Venoms and/or poisons are used for predatory and defensive purposes and have evolved independently in a wide phylogenetic range of organisms, including snakes, spiders, scorpions, and jellyfish, among others [14,15,16]. Ophidian venoms are innovative sources found scattered throughout the animal evolutionary tree [17]. The bioactive properties of these toxins are key points for the discovery of new drugs for possible human therapies [18]. Numerous molecules with different therapeutic approaches have been discovered due to the great biological diversity that is capable of providing us with various solutions to public health problems [19].

It has been scientifically determined that there is a direct correlation between the level of biodiversity within a biome and the complexity of chemical–ecological interactions among organisms occupying the same niche. Furthermore, this biodiversity directly contributes to an increased abundance of diverse molecules and pharmacological activities within these cohabiting organisms. Consequently, it is widely acknowledged that the likelihood of successful drug discovery from natural sources is significantly enhanced when maximal levels of both biodiversity and chemical diversity are present [20].

A specific group of molecules derived from animal venom, which has consistently garnered attention, consists of bioactive peptides. These molecules exhibit high selectivity for specific cell receptors and target specificity, alongside structural stability in bodily fluids and suitability for genetic and synthetic engineering. Due to these characteristics, natural venom peptides serve as promising frameworks for the development of biopharmaceuticals or biotherapeutics. Examples of such peptides include cysteine-stabilized and linear helical peptides [21,22,23].

**Figure 2:**
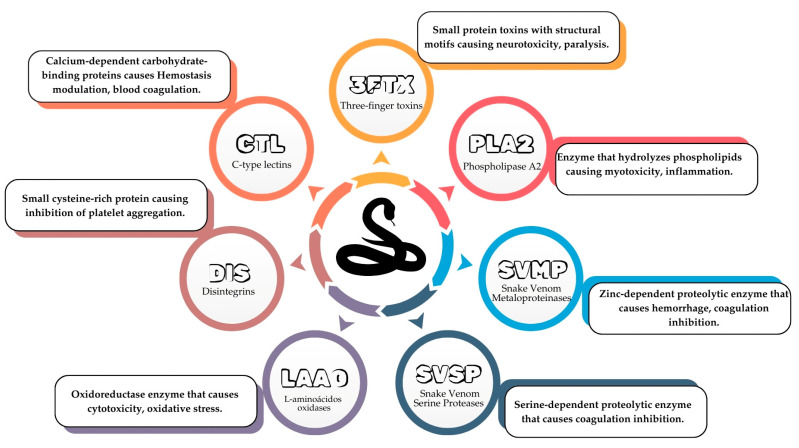
Components, compositions, and the pathological effects of snake venom.

In venom, the primary enzymatic classes are oxidases and hydrolases [24]. Snake venoms contain various compounds, mostly protein mixtures including phospholipase A2 (PLA2) that can cause myotoxicity and inflammation; snake venom metalloproteinases (SVMPs) with pathological effects like hemorrhage and coagulation inhibition; snake venom serine proteases (SVSPs) that can cause coagulation inhibition; L-amino acid oxidases (LAAOs) causing cytotoxicity and oxidative stress; disintegrins (DISs) causing inhibition of platelet aggregation; and C-type lectins (CTLs) causing hemostasis modulation and blood coagulation [25,26]. Three-finger toxins (3FTxs) (that cause neurotoxicity and paralysis), PLA2s, SVMPs, and SVSPs are the most abundant components of these venom protein families, which exhibit multifunctionality and are encoded by multilocus gene families [27,28]. Figure 2 summarizes the composition and pathological effects of these components.

These snake venom components play a crucial role in snakebite accidents and have been adopted in various therapies, including anticancer agents, antihemorrhagic agents, and antihypertensive agents [24,29,30,31]. As an example of an antihypertensive agent, there is the contemporary pharmaceutical known as captopril which was synthesized using the venom obtained from a type of pit viper that is endemic to Brazil, scientifically referred to as *Bothrops jararaca*, commonly known as the Brazilian lancehead snake [32].

The pathological effects of snakebites are diverse and can include neuromuscular paralysis (neurotoxicity), hemorrhage and coagulopathy (hemotoxicity), and/or local swelling, blisters, and tissue necrosis (cytotoxicity) around the bite site [4]. Most venoms of the Viperidae family and some Elapidae induce local tissue damage. The PLA2 present in these venoms is mainly responsible for the myotoxic action leading to myonecrosis by binding to the plasma membrane of muscle fibers, causing its integrity to rupture [33,34].

The development of Small Molecule Therapeutics (SMT) for the initial and adjunctive treatment of snakebites has been shown by Bulfone et al. [35] and these types of review studies are related to enabling successful strategies and potential uses such as drug targets on venom molecules.

## 3. Snake Venom as Treatment

As venoms are a cocktail mixed with toxins with plenty of physiological activities, some products from snake venoms are available for a range of disease treatments. As cited before, captopril is an example of an antihypertensive agent, acting as an angiotensin-converting inhibitor, produced from research on bradykinin potentiator peptides (BPPs). Snake *Bothrops jararaca* venom’s BPPs aid in capturing the target by lowering its blood pressure. In pharmaceuticals, captopril is used in a similar manner for humans: it relaxes blood vessels, which is indicated for hypertensive patients [16,32,36,37].

Eptifibatide, a synthetic cyclic heptapeptide disintegrin, used to prevent the formation of blood clots, is a peptide derived from the *Sistrurus miliarius barbourin* snake, a rattlesnake species [38,39]. Tirofiban, inspired by the echistatin structure, also a short monomeric disintegrin from the venom of the snake species *Echis carinatus*, is also a blood thinner; similar to Eptifibatide, this medicine can also prevent the formation of blood clots in the medical condition that leads to acute blood blockage of blood flow to the heart and heart attack [38,40].

Another important medicine produced from snake venom is Batroxobin, isolated from *Bothrops moojeni* snake venom. Its pathway includes the dissolution of fibrinogen to fibrin and D-dimer and the mobilization of endothelial cells to promote thrombolysis [41]. In nature, it enables the snake to capture the prey by activating blood clotting. Clinically, this capability was explored to treat different types of bleeding and thrombosis [16].

A PLA_2_ from *Bothrops erythromelas*, an endemic snake in northeast Brazil, was isolated, and its biological activity was tested confirming antibiofilm activity against *Acinetobacter baumannii* and antibacterial activity against *Staphylococcus aureus* [42,43].

Still under research are compounds from snake venoms such as Collinein-1, crotamine, and Crotalphine, which have shown potential for some clinical disorders. First, Collinein-1 is a serine protease from *Crotalus durissus colllineatus*, a species that occurs all over Brazil as well as in other countries in South America. This toxin presents the ability to block a specific potassium channel expressed in some cancer lines, reducing the tumor cells’ viability [11,37,44]. Second, crotamine, a compound from the venom of the *Crotalus durissus terrificus* sp., widely distributed in Brazil [44], is a peptide with the ability of membrane translocation with cytoplasmic, vesicular, and nuclear distributions. In melanoma and lymphoma cells, it can undergo an intake process during cell proliferation, constituting a potential anticancer drug [37,45,46,47]. Crotalphine, also isolated from the snake *Cortalus durissus terrificus* venom, is a peptide that has shown activity against peripheral opioid receptors and has been investigated as a potent painkiller for cancer pain [47,48,49].

In antivenom serum, IgG is fractionated to produce F(ab) and F(ab′)2, which reduces adverse reactions and increases efficacy. Commercial antivenoms constitute polyclonal mixtures of antibodies or their fractions raised against all toxin antigens in a specific venom. Improvements in antivenoms aiming for safety and more effectiveness have taken place in recent years. Proteomics and transcriptomics are two of these improvements that have been applied to venom toxin composition (venomics), guiding the understanding of medically important toxins. Also, it is important to point out that these methodologies can identify toxins that contain epitopes recognized by antivenom molecules (antivenomics). Furthermore, humanized and fully human monoclonal antibodies and their fractions as well as enzyme inhibitors have been experimentally developed against venom toxins [50].

## 4. Challenges in Venom Therapy: Addressing Formulation Needs and the Role of Nanotechnology

Venom composition has been studied and it has been demonstrated that it is composed of 100 to 500 pharmacologically active compounds. As shown, there are millions of natural products that can be used for drug discovery. Nevertheless, less than 0.01% have been identified and characterized and a large proportion of toxins act on unknown receptors. Well-known toxins, usually, are described incompletely; the reasons for this are the difficulties in obtaining reliable sources of venoms, inadequate use of screening tests, difficulties in purifying and characterizing the given toxin in detail, and the limited number of academic or industrial research groups working, which is increasing significantly [51,52].

Although there is a larger number of molecules in the venom pool to be analyzed, multiple sophisticated and complementary bioanalytical methodologies and approaches are employed for the isolation, characterization, and assessment of the pharmacological attributes of proteins and peptides found in snake venom. These efforts are directed towards advancing drug discovery investigations. Purification of the peptide requires a combination of chromatographic methods, and, recently, a new online microfluidic high-resolution screening method was employed to identify neurotoxic components [53]. Search-based sequencing uses bioinformatics tools, such as Mascot (Matrix Science) or Protein Pilot (ABSciex), modern mass spectrometers (MSs), and ESI or MALDI TOF mass spectrometers. MS/MS, (MS)_n_, or cleaving of the peptide backbone is performed using a variety of methods, including post-source decay (PSD), collision-induced dissociation (CID), and high collision energy dissociation (HCD). The radical fragmentation technique is used to analyze peptides with labile posttranslational modifications, such as electron capture dissociation (ECD) and electron transfer dissociation (ETD) [54]. As shown briefly, a considerable number of techniques are required to guarantee an optimal characterization of animal toxins.

Other approaches can be explored in the field of mass spectrometry with direct infusion nanoelectrospray ionization (nano-ESI-MS) to evaluate peptide distributions in *Bothrops* snake venoms, as shown by Souza et al. [55], or even the use of proline present in *B. jararaca* venom, as carriers of therapeutic agents, which cannot cross the cell membrane, increasing their applicability in drug delivery [56].

**Figure 3:**
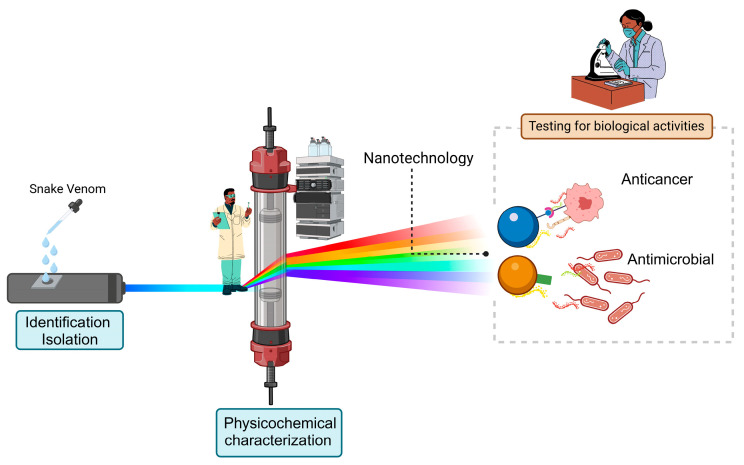
Representation of snake venom characterization and evaluation for biological tests.

Furthermore, identification, isolation, and purification are not the only concerns in this field nowadays, but the therapeutic potential of these venoms has also been considered, e.g., as shown in Biswas et al. and Munawar et al. [57,58]; this method is illustrated in Figure 3.

According to Biswas et al. [57], nanosized particles exhibit unique properties that can be harnessed to enhance the pharmacological and therapeutic efficacy of medications. The process of nanoencapsulation for these potent therapeutic compounds serves not only as a platform for improved drug delivery but also contributes to heightened stability, increased bioavailability, and targeted application of the drugs. While larger molecules may be subject to elimination from the body, nanoparticles, due to their size, are effectively taken up by cells. A variety of hydrophilic nanoparticles, including chitosan, nano-gold, nano-silver, magnetic and superparamagnetic nanoparticles, and dendrimers, among others, are currently under extensive investigation for their role as drug delivery vehicles. This involves the conjugation of these nanoparticles with various therapeutically potent substances, including venoms and toxins, particularly peptides, proteins, and antigens.

## 5. Commonly Used Nanosystems to Encapsulate Snake Venom

Nanomedicine emerges as a pioneering field that holds great promise for the twenty-first century, employing nanotechnology to enhance healthcare and pharmaceutical products [59,60]. Consequently, it has been determined that diverse nanocarriers present numerous benefits, including (i) ensuring consistent drug dosage and targeted delivery while avoiding concentration fluctuations, (ii) maximizing therapeutic efficacy while minimizing potential side effects and risks of toxicity, and (iii) providing drug protection against enzymatic degradation [61].

**Figure 4:**
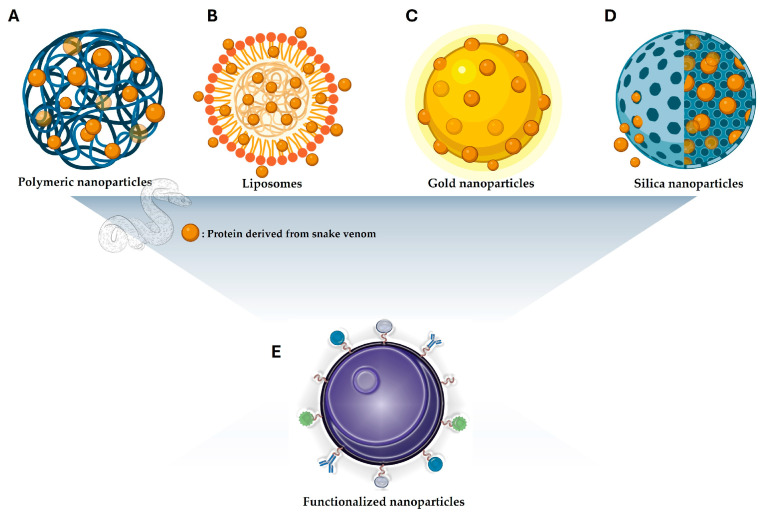
The use of nanotechnology is based on the incorporation of proteins derived from snakes. (**A**) Polymeric nanoparticles; (**B**) liposomes nanoparticles; (**C**) gold nanoparticles (GNPs); and (**D**) mesoporous silica nanoparticles. (**E**) Schematic representation showing that nanoparticles, regardless of their nature, can be modified, thus improving their physical, chemical, and biological properties.

In the field of drug delivery systems, nanomaterials have emerged as key contributors to enhancing drug stability, solubility, release control, minimizing toxicity, and achieving enhanced therapeutic effects [62]. Various nanocarrier systems (Figure 4), including polymeric nanoparticles, inorganic nanoparticles, and nanohydrogels, have been developed to address these challenges [63].

The progress made in the field of nanotechnology, specifically in nanovaccinology, has mitigated the limitations associated with conventional vaccines and paved the way for the creation of an innovative nanoparticle-based delivery system for vaccines. Nanotechnology allows designing nanoparticles with varying characteristics such as composition, size, shape, and surface properties to meet specific medical needs [64].

Currently, there is a significant research trend using nanotechnology in venom and toxin studies [65]. The research on nanotechnology and snake venom/toxin has two distinct dimensions: (i) the use of nanotechnology in developing antivenom for snake venom and (ii) the application of nanotechnology in developing drug indicators against physiopathologies such as arthritis, cancer, and HIV. So far, antivenom snake serum (ASVS) has been the only clinically used antidote against snakebite, which was developed in 1894 at the Pasteur Institute in Paris by Dr. Calmette. The limitations (cost factor, shelf life, no protection against local effects, hemorrhage, and organ toxicity) and side effects (anaphylactic shock, pyrogenic reaction, and serum sickness) on patients are well known [66].

Snakebites can be treated by the administration of antivenom serum and can be associated with adjuvants such as immunoadjuvants that increase the immune response to antigens which are unable to stimulate the immune system with their properties [67]. In this sense, the use of nanotechnology has been investigated aiming at inducing effective adaptive immune responses and reducing tissue damage caused by the toxins present in the venom [68]. In addition, these toxins can be evaluated for their therapeutic efficacy, against emerging diseases [69]. Approaches using nanotechnology emerge as trends and strategies in current medicine.

With a wide range of applications in nanotechnology in biomedical sciences, it is essential to understand how these nanoparticles function within the biological system. It is a huge commercial market today, with billions of investments in global prospects, and it is expected to be a giant market in the future. Various nanoproducts are already commercially available, and numerous products are under research and development. Therefore, nanotechnology can be considered the next technological revolution that has a direct impact on human health and the environment [66].

Over the years, snake venoms have been used as medical research tools or therapeutic/diagnostic agents [57,70]. Calmette et al. [71] proposed that the physiologically active components of these venoms may have therapeutic potential. They demonstrated that these venoms could kill cancer cells in animal models. Subsequently, many reports established the anticancer potential of different species of Elapidae, Viperidae, and Crotalidae snake venoms [72,73,74]. It has also been observed that these compounds are highly toxic, producing adverse neurological, cardiac, and respiratory effects [75].

Many attempts have been made to increase the therapeutic potential and decrease the toxicity of venom toxins in biological systems. One such approach in modern research has been nanotechnology, which can provide higher efficacy and lower toxicity [66]. A common approach is to attach the protein present in the venom to a nanoparticle through the process of non-covalent electrostatic adsorption [76]. However, this non-covalent interaction is not stable at high salt concentrations and pH values above the protein’s isoelectric point. A covalent bond between the toxin protein of the venom and the nanoparticle is more stable, and the binding site to the nanoparticle can be controlled. Conjugation of nanoparticles with snake venom can cause protein folding/unfolding [77]. It is advised to verify the function of the peptide/protein structure after conjugation with nanoparticles. Co-functionalization of nanoparticles with polyethylene glycol can aid in protein unfolding and can be used in the formation of nanoparticles loaded with snake venom [66].

In the pharmaceutical field, there are several approaches involving polymers, and one alternative is poly(ε-caprolactone) (PCL), which has been used as a material for controlled release to produce micro- and nanospheres, reducing toxicity and increasing hydrophobic solubility and passivation, segmentation, or action [78]. PCL’s high permeability to various drugs and molecules allows them to be released from the matrix by diffusion [79,80].

The biodegradability of PCL has garnered considerable attention in academic and research circles [81,82]. Numerous research investigations have documented the effective utilization of multi-arm PCL-based nanoparticles in conveying a spectrum of medications, encompassing anticancer agents, antibiotics, and anti-inflammatory substances [83,84]. Colloidal systems utilizing PCL have sparked significant interest owing to their application as drug delivery vehicles [85]. Self-assembled structures such as micelles have been extensively investigated for their efficacy in encapsulating a diverse array of drugs and proteins [86].

The parameters of each technique must be adjusted to obtain stable spheres with sufficient electric charge and solubility and with the smallest diameter. These factors are important because they determine the drug target in the body, the type of cellular interaction, and its release [87].

## 6. Encapsulation of Venom Fractions with Bioactivity and Its Applications

### 6.1. Nanosystems Containing Bothrops Snake Venom

In the context of improving antivenom serums, nanoparticles are colloidal dispersions that can be targeted through a modified release of protein biomolecules [88]. In a study by Santos-Silva et al. [68], a new immunoadjuvant was prepared based on cationic PLGA nanoparticles (CNps), which are biocompatible with *Bothrops jararaca* venom, thus comparing antibody production with a conventional immunoadjuvant such as aluminum hydroxide. The particles showed positive charges by the addition of polyethyleneimine (PEI), where an increase in zeta potential was observed with an increasing PEI/polymer ratio. The concentrations of 0.5 and 1.0% were used for *B. jararaca* (BJ) venom in the CNps, obtaining an encapsulation efficiency higher than 98%. In the presence of venom, the nanoparticles showed little variation in size, given in nanometers, for CNps without the venom (140.0 ± 1.8), CNps + 0.5%BJ venom (167.3 ± 6.3), and CNps + 1.0%BJ venom (168.7 ± 3.8), and zeta potential, given in millivolts, as 47.38 ± 6.24, 42.83 ± 14.6, and 35.6 ± 13.4, respectively, with more than 98% encapsulation efficiency for both concentrations. From the results in the in vivo studies, it was observed that, in the immunization protocol, animals treated with only CNps did not show an immune response, whereas when treated with aluminum hydroxide or nanoparticles containing the venom, they produced detectable antibodies at the lowest dilution tested (1 to 51,200); no statistical difference was shown between aluminum hydroxide and CNps, making cationic nanoparticles promising in the study of nanoformulations containing bioactive molecules as immunoadjuvants.

Among the nanosystems related to the *Bothrops* genus, nanoparticles are found in different studies, such as the one using chitosan, which is a polysaccharide obtained by partial N-deacetylation of chitin and has also been investigated as an immunoadjuvant for reducing side effects and improving the efficiency of serums and vaccines [89,90]. Thus, CNPs loaded with venoms of *B. jararaca* and *B. erythromelas* were evaluated by Soares et al. [67] for the production of serum against these venoms. According to these authors, the particles were obtained by the ionic gelation technique, through titrations of a tripolyphosphate (TPP) in a chitosan solution, with an average size of 167.5 nm, zeta potential (ZP) of +24.5 mV, and polydispersity index (PdI) of ≤0.3. To incorporate the proteins in the formulation, they were carried in the particles through the incorporation method using a TPP solution, causing no harm to the formation of CNPs. The encapsulation efficiency showed values of 87% for all formulations tested containing *B. erythromelas* venom. For *B. jararaca*, the protein encapsulation efficiency reached levels higher than 67%. The results showed that chitosan is a less inflammatory biopolymer that requires a lower dose of antigen, with equivalent or better performance than aluminum hydroxide as an immunoadjuvant.

Another applicability for bioactive components presents in snakes of the genus *Bothrops* is in the development of drugs with bacterial resistance. In this sense, *Bothrops jararacussu* venom-loaded CNPs were studied by Ribeiro et al. [91] with potential antibacterial activity. The nanoparticles were obtained by ionic gelation, with a size of approximately 160 nm (PdI < 0.5) and a protein loading efficiency higher than 70%. The antibacterial activity was performed by broth microdilution on *Escherichia coli* and *Staphylococcus aureus* strains. *Bothrops jararacussu* venom showed antimicrobial activity and the nanoparticle containing 0.5 and 1.0% of venom showed great potential as an antibacterial agent against Gram-positive strains.

Another approach to the utilization of *Bothrops* venoms is related to the induction of oral tolerance through the previous oral administration of an antigen, as seen in the work of Tsuruta et al. [92], who evaluated the induction of tolerance to snake venoms adsorbed to/encapsulated in nanostructured SBA-15 silica in BALB/c mice, observing specific tolerance and cross-reactivity with other toxins of *Bothrops* species. The authors developed a protocol in BALB/c mice for oral tolerance, by administration of *B. jararaca* venom, measuring antibody titers in antisera after immunization with snake venoms, to understand the mechanisms involved. With this, it was observed that the induction of oral tolerance by the venom will depend on the dose administered and is specific for each snake species, showing that tolerance was induced more effectively when the same snake venom was used in oral administration and immunization, even when the antiserum showed cross-reaction with venom from other snakes. The study of Wambre and Jeong [93] demonstrates the application of oral tolerance as a non-invasive form, allowing studies in the prophylaxis and treatment of diseases.

Nanotechnology also allows for a reduction in the toxicity of venoms and for use in the immunization of horses for the development of antisera to treat snakebites, reducing the stress and suffering of the horse, as reported by Costa et al. [94], who proposed a modification of the immunization protocol, with inactivated venoms and encapsulation in stabilized liposomes. The particles showed an encapsulation efficiency of 59% to liposomes with venom of *Bothrops* (LB) and 99% to liposomes with crotalic venom (LC), as followed by filtration on Sephacryl S1000. Light scattering measurements led them to conclude that both LB (119 ± 47 nm) and LC (147 ± 56 nm) were stable for 22 days at 4 °C, even after lyophilization. The venoms used were from five different *Bothrops* species (*B. alternatus*, *B. jararaca*, *B. jararacussu*, *B. moojeni*, and *B. neuwiedi*) and *Crotalus durissus terrificus*, which were solubilized in an MPB-pBB/EDTA buffer (20 mM phosphate, 295 mM mannitol, 5 mM EDTA, 3 mM pBB, pH 7.2). Therefore, the aim of this study was to inactivate the venom in order to reduce the toxicity and inhibition of PLA_2_ resulting in the stabilization of liposomes, using the chelating agent CaNa_2_EDTA and EDTA to increase the inhibition of PLA_2_. The authors also pointed to the importance of formulation stability after freeze-drying, which was demonstrated in another study by Oliver et al. [95]: freeze-dried liposomes containing PLA_2_ and partially rehydrated at controlled humidities did not show PLA_2_ activated during freeze-drying but were more active during rehydration. Another problem found was the stability of the membrane after freeze-drying, which was overcome by using mannitol as a cryoprotectant. Bothropic venom-loaded liposomes showed an efficiency of 99%, while crotalic venoms showed 59%. After the procedures, no signs of venom toxicity were observed in the horses immunized with the liposomal formulations containing snake venom, due to venom alkylation and delivery within stable nanosystems, showing no differences in their reactivity. In addition, the lipolytic activity associated with venoms is directly related to the PLA_2_, which can induce membrane rupture through fusogenic agents such as lysophosphatidylcholine [96,97,98].

According to Carvalho et al. [99], another possibility for the use of liposomes is the use of sphingomyelin in its composition for the encapsulation of *B. jararaca*, resistant to phospholipase, with a high cholesterol content to increase membrane stability. The authors used a mouse model to evaluate the toxicity of *B. jararaca* venom encapsulated in liposomes, in order to protect animals against the lethal effects of poisoning. The encapsulation method was dehydration–rehydration vesicles according to Kirby and Gregoriadis [100]. Two immunostimulants were used: a hydrophilic lipopolysaccharide (LPS), which was solubilized together with the venom, and hydrophobic monophosphoryl lipid A (MPLA), solubilized with the lipids. There was a reduction in the clinical toxicity of encapsulated *B. jararaca* (BJ) venom, where no mice died after injection of a dose equivalent to 6×LD 50 of liposome-encapsulated BJ venom. The formulations were stable at 37 °C, with a retention of 65% of the originally encapsulated *B. jararaca* venom, allowing future studies to examine the use of liposomes as immunoadjuvant systems for commercial antivenoms.

The PLA_2_ isolated from *Bothrops jararacussu* venom can also be evaluated for its anti-*Leishmania amazonensis* activity in vitro according to de Barros et al. [101] who added 2 mg/mL of the PLA_2_ to a lipid mixture, which was solubilized in chloroform and dried under nitrogen flow, with the formation of the vesicles by the extrusion method. The liposomes showed monodisperse populations with 205.2 nm of the encapsulated Asp49 liposomes. The zeta potential was −25.4 mV and there was an encapsulation rate of 69.6%, meaning 1.4 mg of the toxin was detectable in 1 mL of the liposomes produced. The formed nanosystems reduced 78% of promastigote forms and preserved 82% of J774 macrophage viability. In addition, there was a considerable reduction (55%; *p* < 0.05) in amastigote forms, compared to the control group, conferring the possibility of biotechnological studies using nanotechnology against neglected diseases, such as leishmaniasis. The same liposome nanosystem was later used by the same research group of de Barros et al. [102] in another study to evaluate the in vivo anti-*Leishmania amazonensis* activity. In comparison to the control group, a 57.1% reduction was observed in the paw tissue. The infected groups treated with Asp49-PLA2 liposomes exhibited a notable increase in TNF-α production in the lymph nodes and paws (43.73 pg/mL ± 2.25 and 81.03 pg/mL ± 5.52, respectively), along with elevated nitrite levels (31.28 μM ± 0.58 and 35.64 μM ± 5.08 in lymph nodes and paw tissues, respectively) when compared to the untreated groups.

### 6.2. Nanosystems Containing Crotalus Snake Venom

Chitosan nanoparticles were developed by Glaucia-Silva et al. [103] through an ionic gelatinization method containing *Crotalus durissus cascavella* crude snake venom capable of inducing murine immunization, incorporating auxiliary components to enhance the immunogenicity of the serum or vaccine. Chitosan nanoparticles (CNPs) exhibit characteristics such as biocompatibility, biodegradability, mucoadhesive properties, and controlled drug release. Physicochemical properties of these CNPs were determined without venom (size: 155.5 ± 7.6 nm; PdI: 0.296 ± 0.007; zeta potential: +30.8 ± 12.9 mV), with 0.5% of *C. d. cascavella* venom (size: 147.4 ± 9.0 nm; PdI: 0.316 ± 0.004; zeta potential: +36.0 ± 18.6 mV), with 1.0% of *C. d. cascavella* venom (size: 147.7 ± 12.0 nm; PdI: 0.323 ± 0.009; zeta potential: +41.80 ± 14.1 mV), and with an encapsulation efficacy over 95% for both concentrations. Immunization with CNPs incorporated with *C. d. cascavella* in BALB mice exhibited high effectiveness when compared with the control, producing high IgG titters until the dilution of 1 to 6400, positioning them as a promising pharmaceutical agent.

Crotoxin, a major component of the venom of *Crotalus durissus terrificus*, demonstrated in vivo immunomodulatory and anti-inflammatory characteristics [104]. Thus, in order to nanoencapsulate crotoxin, Sant’Anna et al. [105] employed nanostructured mesoporous silica (SBA-15) and evaluated its responsiveness in the context of experimental autoimmune encephalomyelitis (EAE) in a murine model of multiple sclerosis; SBa-15 was utilized, with the incorporation of crotoxin diluted in PBS at a ratio of 1:10 (toxin to silica) maintained for 24 h at 2 °C to 8 °C with sporadic agitation. It was observed that the use of crotoxin+SBA-15 in the initial phase of development could ameliorate EAE-induced neuroinflammation by preventing the recruitment of peripheral Th17 cells, reducing cellular infiltration into the central nervous system, decreasing IL-17, and inhibiting glial cell activation. This, in turn, led to a reduction in the intensity and incidence of clinical signs while preserving muscular function. Furthermore, it demonstrated potential as a nanocarrier for use with biological compounds, exhibiting significant efficacy in reducing toxicity, allowing for increased dosage without a corresponding rise in toxic effects, and enhancing the delivery and effectiveness of crotoxin compared to its non-encapsulated form [105,106,107].

In a study conducted by Karpel et al. [108], gold nanoparticles (GNPs) were employed to be optimized with crotamine. Crotamine was adsorbed onto GNPs through the interaction involving the covalent bonding of the peptide and a polyethylene glycol (PEG) ligand, resulting in the formation of the PEG–crotamine–GNP transport complex. The particles exhibited a zeta potential close to zero (−0.6 mV) and a crotamine-free surface charge (−25 mV), with diameters for GNPs, GNP–PEG, and GNP–PEG–crotamine measured at 10, 34, and 43 nm, respectively. GNPs associated with crotamine demonstrated high efficiency in being absorbed by HeLa cells, indicating the sustained cell-penetrating nature of crotamine even after complex association. In this context, GNPs are emerging as increasingly significant players in multimodal and multifunctional molecular imaging for the early-stage detection of diseases such as cancer. Moreover, the research highlighted that GNP–crotamine complexes have evolved into potential platforms for therapeutic agents.

In a study using chitosan nanoparticles (CNPs), Jimenez-Canale et al. [109] studied the venom of *Crotalus molossus molossus* for cytotoxic activity against T-47D breast carcinoma cells. The particles, derived from the venom (primarily composed of VAP2A, Ruberlysin, Apoxin I, and PLA2), exhibited dimensions of 415.9 ± 21.67 nm, a polydispersity index (PdI) of 0.44 ± 0.03, and a zeta potential of +28.3 ± 1.17 mV. They displayed distinct features of a smooth and semi-spherical surface. Moreover, these particles demonstrated non-hemolytic properties in human red blood cells and reduced cell viability in T-47D breast carcinoma cells.

Utilizing liposomes as a nanoencapsulated system, Magalhães et al. and Freitas and Frézard [110,111] generated nanoliposomes encapsulating crotoxin to observe the effects of these nanoparticles as immunostimulants and their immunomodulatory impacts for potential antivenom therapy or vaccination purposes, respectively. In both studies, liposomes associated with crotoxin elicited immunization effects in rats against the respective venom, concurrently reducing toxicity and allowing for increased doses with diminished toxic effects. Magalhães et al. [110] showed that the toxicity of liposomes containing crotoxin was influenced by the composition of the membrane. Thus, encapsulating crotoxin in liposomes composed of sphingomyelin significantly diminished its toxicity; even when mice were inoculated with more than 50 times the lethal dose (LD_50_) of crotoxin, none succumbed. When crotoxin was encapsulated into dehydration–rehydration vesicles, a protection level exceeding 50% was achieved for both Magalhães et al. and Freitas and Frézard [110,111]. Immunization with 100% protection was achieved by Magalhães et al. when the immunization with liposomes encapsulating crotoxin was prepared using a non-phospholipid mixture and without immunostimulants, resulting in this intriguing outcome. This method proved to be cost-effective, with a preparation cost of approximately USD 80 per liter of liposome preparation.

By employing the Stöber method, Baudou et al. [112] investigated the adsorption of *Crotalus durissus terrificus* snake venom onto silica nanoparticles (SiNPs), resulting in the formation of nanovenoms. Through experimentation with various enzymatic activities, the researchers demonstrated that SiNPs effectively bound snake venom proteins while preserving their biological activity. This study demonstrated that venom nanoparticles conserve the cytotoxic effect that was tested in THP-1 cells (human monocytic cell line derived from acute monocytic leukemia), and a significant decrease in proliferation (∼84%) was constated in different concentrations tested for nanoparticles venom (5 and 10 μg/mL).

Improved biodistribution is observed when the venom is encapsulated. Gomes et al. [113] conducted a comparative study on the biodistribution of free and encapsulated *Crotalus durissus terrificus* venom in mice. Upon subcutaneous inoculation of the encapsulated venom at a higher dose of 25 μg/mouse (2 LD_50_), no *C. d. terrificus* was detected in the kidney, spleen, brain, or other tissues. On the other hand, free *C. d. terrificus* venom following subcutaneous injection was detected in the serum 15 min after administration, reaching its peak concentration at 30 min (45 ± 5 ng/mL), and was cleared from circulation within 6 h. Two hours post-inoculation, the venom was found in the kidney (57 ± 9 ng/g of tissue), spleen (18 ± 4 ng/g of tissue), and brain (14 ± 6 ng/g of tissue). However, after intravenous injection of the encapsulated venom at a dose of 5 μg/mouse (LD_50_), venom was observed in liver and spleen tissues. Whether administered subcutaneously or intravenously, free *C. d. terrificus* venom was initially detected in the kidney. The paper focuses on the implications of encapsulated venom biodistribution in relation to the reduction in toxicity and enhancement of adjuvanticity effects. All the information about the nanosystems used to encapsulate the different snake venoms is summarized and highlighted in Table 1. Some nanosystems of Elapidic venom can also be found in the literature.

### 6.3. Nanosystems Containing Lachesis Snake Venom

We conducted a comprehensive search across various available databases aiming to identify studies that explored the utilization of *Lachesis* snake venom in nanosystems. However, no studies reporting this specific association have been found thus far. The absence of identified studies highlights a current gap in scientific knowledge regarding the utilization of *Lachesis* venom in nanotechnology. This finding underscores the need for future research in this area to explore the potential of this association and its possible applications in nanomedicine and nanotherapy.

To the best of our knowledge so far, it is noteworthy to mention that although no studies investigating the combination of *Lachesis* venom with nanotechnology were found, in our search, there exist numerous studies reporting on the biological activities of this venom. These studies have elucidated various pharmacological and therapeutic properties associated with *Lachesis* venom. Future research endeavors could draw upon these biological activities as a foundation for bridging these two scientific domains, thus exploring the potential synergistic effects and novel applications that may arise from integrating nanotechnology with the known biological activities of *Lachesis* venom.

In the realm of *Lachesis* venom’s biological activities, several studies have shed light on its remarkable properties. For instance, research by Stransky et al. [114] demonstrated the potent cytotoxic effect of different cell lines, the toxicity observed in this context encompasses the integration of diverse mechanisms of cell death. *Lachesis muta muta* venom decreased, in a concentration-dependent manner, the cell viability of the tumor (MGSO-3 and HeLa), as well as immortalized (VERO, EA.hy 926) and normal cells (keratinocytes). Different levels of toxicity were observed for each cell type. Vero cells (EC_50_ = 0.83 μg/mL) and MGSO-3 (EC_50_ = 2.26 μg/mL) were the most sensitive to venom and HeLa cells (EC_50_ = 7.14 μg/mL) were more resistant.

Additionally, studies by Fuly et al. [115] highlighted the procoagulant, proteolytic, and phospholipase A_2_ activities. Furthermore, according to Almeida et al. (2015) [116], investigations on patent application fields showed anti-aging antioxidant preparations [application number: RU20010122763], prevention and treatment of viral and bacterial infections in avian species [application number: RU20020111635], antimicrobial agents [application number: IE20070000737, WO2008EP08602], and analgesic composition [application number: US2015030691]. These notable findings not only underscore the diverse biological activities associated with *Lachesis* venom but also serve as a foundation for future studies that can harness these activities in tandem with nanotechnology, opening new avenues for therapeutic interventions and biomedical applications.

## 7. Theragnostic Applications of Polymeric Nanosystems

Crotamine exhibits distinct selectivity towards actively proliferating (AP) cells [117,118,119] and is primarily internalized via endocytosis, even when loaded with a cargo [120]. It selectively facilitates the delivery of plasmid DNA into AP mammalian cells [118,120]. The capacity to transport cargo without requiring covalent bond formation [120], coupled with its preferential cytotoxicity towards tumor cells [121], indicates the potential utility of crotamine in cancer therapy and/or diagnostics.

Short peptides derived from crotamine, such as CyLoPs (Cytosol Localizing Peptides) and Nucleolar Targeting Peptides (NrTPs), have been extensively studied [122]. Notably, NrTP-1, a prototype of the NrTP series, was originally designed [123] and subsequently modified by replacing the cysteine residue at position 4 with a serine, resulting in an analog called NrTP-6 (YKQSHKKGGKKGSG) [124,125]. NrTP-6 has demonstrated efficient penetration and accumulation in specific cancer cell lines and populations, making it a promising theragnostic probe for imaging cancer heterogeneity and targeted delivery of chemotherapeutics to specific subpopulations of cancer cells [126].

Crotalicidin, a vipericidin [127], and an Asian elapid CRAMP (cathelicidin-related antimicrobial peptide) have exhibited notable biological activities, particularly in relation to their antipathogenic and antitumoral properties, as extensively reviewed elsewhere [128,129,130]. The interaction of crotalicidin and elapid CRAMPs with biological membranes and biomimetics has been extensively demonstrated, encompassing not only the native, full sequences but also short derived peptide fragments. Their ability to induce perturbations in the plasma membrane is closely associated with the mechanisms underlying their antimicrobial activity [131,132,133,134,135], as well as their pronounced antitumor effects, which have been experimentally validated in vitro using various cancer cell lines [136,137].

Cardiotoxin VII4, obtained from the Mozambique spitting cobra *Naja mossambica mossambica*, has been identified as a cell-penetrating peptide with the capability to enter mitochondria. Its application led to mitochondrial fragmentation in both mouse primary cortical neurons and human neuroblastoma (SH-SY5Y) cells [138]. A short cationic [13-mer] peptide derived from the C-terminal region of the Lys49 PLA2 from the venom of the broad-banded copperhead snake *Agkistrodon contortrix laticinctus* was prepared and this imparted engineered peptide characteristics of CPPs and AMPs and revealed an effective leishmanicidal activity [139].

According to Sciani et al. [56], Bradykinin-Potentiating Peptides (BPPs) derived from the venom of *Bothrops jararaca* have exhibited notable cell-penetrating capabilities. Fluorescence microscopy experiments revealed the translocation of these peptides into the cytoplasm of human melanoma tumor cells (SK-MEL-28 and A2058), without any detectable cytotoxic effects even when 4 µM peptide was used. Thus, no change in metabolism, cell cycle, or morphology was observed with either labeled or unlabeled CPPs, clearly demonstrating their role as cargo carriers rather than drugs themselves. A label-free method for identifying CPP peptides, regardless of biological source, has been developed and validated, potentially opening the way for the discovery of new and more effective drug delivery systems.

## 8. Clinical Trial, Patents, and Market

Increasingly sustainable economies are being represented by the bioeconomy. The focus is on bio-innovations that explore renewable natural resources for bio-inputs crucial to the development of new bioprocesses and bioproducts [140,141]. The enhancement of bio-entrepreneurship [142] with biotechnology plays a pivotal role in shaping this new knowledge-based bioeconomy [143,144,145]. Innovative products emerge from the integration of cellular and molecular biotechnology techniques with bio-resources [146]. In this context, both known and yet-to-be-discovered organisms that make up our natural biodiversity could serve as primary sources for novel bio-compounds with boundless applications in what is nowadays called One Health.

An example of bioactive compounds that have been used in the design of new therapeutic agents and products with diverse pharmacological applications, cosmeceuticals, and diagnostic tools can be found in a review carried out by [11].

A considerable number of patents applied to the discovery of medicines based on bioactive compounds from venomous animals have been developed [116]. New technologies, such as nanotechnology, have been used for the targeted delivery of medicines based on the delivery of materials from synthetic and natural sources to their clinical applications, aiming to improve pharmacokinetics and pharmacodynamics, which is a very prosperous area for the development and study of new products [62].

Snake venom is a rich source of biological compounds. Patents referring to toxins present in the genus *Bothrops* are related to hypotensive and vasodilatory formulations, medicines for the treatment of systemic–degenerative diseases, modulators of acetylcholine receptors, anticoagulant and thrombolytic formulations, bactericides, and others [116]. The captopril was developed based on the bradykinin potentiating factor obtained from *Bothrops* jararaca snake venom and it was the first animal toxin-based drug approved for human use [147,148].

Almeida and collaborators [116] report that *Crotalus* genus snakes are the most investigated in Brazil for drug discovery, and patent registrations for bioactive compounds originating from the venom of this type of snake are used to obtain various pharmaceutical products with different applications, such as antimicrobial drugs, drugs for cancer treatment, analgesic drugs to treat AIDS, anti-inflammatory agents, and others. A study developed by Boldrine-Franca et al. [149] demonstrated that a toxin isolated from the venom of the snake *Crotalus durissus collilineatus* demonstrated important activity against several strains of cancer, being a potential candidate for the development of a pharmacological product.

The United States Patent and Trademark Office [150] stated, by a cooperative patent classification (CPC), that patents including snake venom are one of the most frequently reported patents, with 7242 patents reported until the year 2020 in the field of agriculture [151].

Clinical trials using snake venom are challenging due to their complex protein and peptide toxins, making them clinically challenging and scientifically fascinating [152]. High variability in snake venom composition is responsible for the various clinical manifestations in envenoming, making it difficult to find clinical trials using snake venom [4]. Clinical trials using snake venom are difficult to find due to the lack of relevant studies in the literature [153,154]. However, a recent review by Matkar et al. highlights the significance of research on natural products, emphasizing the role of serine proteases and their applications in human therapies, including amyloid formation [155]. This underscores the critical need to explore the diverse therapeutic potential of natural product-derived compounds, particularly those sourced from animals, when starting new clinical trials.

## 9. Conclusions

The application of snake venom in medical therapies has garnered global attention due to its potential to advance our understanding of biological activity and enhance immunization strategies against toxic effects. Among the most promising developments is the utilization of polymeric, lipidic, or metal nanoparticles to encapsulate and/or incorporate venom toxins from various snake species. This innovative approach offers significant therapeutic potential by improving treatment efficacy and reducing the damage caused by envenomation.

Despite these advancements, the vast diversity of snake species and the intricate composition of their venoms highlight the substantial knowledge gaps that remain. Continued exploration, particularly focusing on less studied species, is essential to deepen our understanding of venom components and their therapeutic applications. This research not only aims to refine treatments for snakebite envenomation but also opens new avenues for the development of novel therapies. Ultimately, these efforts contribute to expanding scientific knowledge and advancing human health in multiple domains.

## Figures and Tables

**Table 1:** Overview of snake venom and nanobiotechnology-derived nanobiosystems being used for biological application and its nanosystem types and sources.

Molecule/Toxin	Classification/Source	Nanosystems	Characterization Size (nm); PDI; PZ (mV)	Encapsulation Efficiency (%)	Biological Activities	References
Whole venom	*Bothrops jararaca* (BJ)	Cationic PLGA nanoparticles (CNps) +1.0% BJ venom	168.7 ± 3.8 nm, 0.09 ± 0.01 +35.6 ± 13.4 mV	-	Immunoadjuvant	[68]
Whole venom	*Bothrops jararaca* and *Bothrops erythromelas (Bery)*	Chitosan nanoparticles (CNPs)	BJ 10%: 174.7 ± 5.0 nm, 0.203 ± 0.07 +24.91 ± 2.91 mV Bery 10%: 160.0 ± 2.3 nm, 0.302 ± 0.01 +19.00 ± 2.76 mV	BJ 67.7 Bery 87.6	Immunoadjuvant	[67]
Whole venom	*Bothrops jararacussu*	CNPs	160 nm Inferior to 0.5	More than 70%	Antimicrobial activity against Gram-positive bacteria	[91]
Whole venom	*Bothrops jararaca*	Nanostructured SBA-15 silica	-	1 µg of venom adsorbed /encapsulated	Oral tolerance induction	[92]
Whole venom	*Pool of venom containing Bothrops alternatus, Bothrops jararaca, Bothrops jararacussu, Bothrops Bothrops moojeni, Bothrops neuwiedi*, and *Crotalus durissus terrificus*	Liposomes with *Bothrops* venom (LB), and with crotalic venom (LC)	LB (119 ± 47 nm) and LC (147 ± 56 nm)	The liposomal efficiencies of protein incorporation were 99% for the bothropic and 59% for the crotalic venoms	Development of antisera to treat snakebites, reducing the stress of the horse	[94]
Whole venom	*Bothrops jararaca*	Liposomes	-	-	Immunostimulants	[99]
PLA_2_ toxin	*Bothrops jararacussu*	Liposomes	Size: 241.9 and 205.2 nm Zeta: −18 mV and −25.4 mV	-	Anti-*Leishmania amazonensis* activity in vitro and in vivo	[101,102]
Whole venom	*Crotalus molossus molossus*	CNPs	Size: 415.9 ± 21.67 nm PdI: 0.44 ± 0.03 Zeta: +28.3 ± 1.17 mV	48.29 ± 3.84	Cytotoxic activity against T-47D breast carcinoma cells	[109]
Whole venom	*Crotalus durissus cascavella*	CNPs + *C. d. cascavella* venom 1.0%	Size: 147.7 nm PdI: 0.323 Zeta: +41.80 mV	98.9	Immunostimulants	[103]
Crotoxin	*Crotalus durissus Terrificus*	Nanostructured mesoporous silica SBA-15	-	-	Immunomodulatory and anti-inflammatory activity in multiple sclerosis	[105]
Crotamine	*Crotalus durissus Terrificus*	Gold nanoparticle GNPs	Size: 43 nm Zeta: −0.6 ± 3.9 mV	-	Cell-penetrating ability	[108]
Crotoxin	*Crotalus durissus Terrificus*	Liposomes	-	-	Immunostimulants	[110,111]
Whole venom	*Crotalus durissus terrificus*	Liposomes	-	-	Biodistribution of free and encapsulated liposomes	[113]

## Data Availability

Not applicable.
